# Multi-walled carbon nanotubes supported Cu-doped ZnO nanoparticles and their optical property

**DOI:** 10.1007/s11051-012-0817-5

**Published:** 2012-03-28

**Authors:** C. S. Chen, X. D. Xie, T. G. Liu, L. W. Lin, J. C. Kuang, X. L. Xie, L. J. Lu, S. Y. Cao

**Affiliations:** 1 College of Physics and Electronic Science, Changsha University of Science and Technology, Changsha, 410114 People’s Republic of China; 2 State Key Laboratory for Powder Metallurgy, Central South University, Changsha, 410083 People’s Republic of China; 3 College of Physics and Microelectronics Science, Hunan University, Changsha, 410082 People’s Republic of China

**Keywords:** Carbon nanotube, Cu-doped ZnO, Composite powder, Optical property, Synthesis

## Abstract

Multi-walled carbon nanotubes (MWNTs)/Cu-doped ZnO composite powders were prepared by co-precipitation method, and were characterized by X-ray diffraction, electron microscopy, fluorescence spectrum, and ultraviolet spectrum. Experimental results show that the MWNTs can be modified by Cu-doped ZnO nanoparticles with hexagonal wurtzite structure after annealed at 450 °C, and the nanoparticle size is about 15 nm. Two ultraviolet (UV) peaks and a green band centered at about 510 nm are observed in the fluorescence spectrum of MWNTs/Cu-doped ZnO composite powder annealed at 450 °C. Furthermore, MWNTs and Cu doping significantly improve the UV absorption ability of ZnO.

## Introduction

Zinc oxide (ZnO) is a wide direct band gap (3.37 eV at room temperature) semiconductor and possesses superior intrinsic properties such as a large exciton binding energy of 60 meV at room temperature, high photocatalytic activity, low cost, and environmentally friendly. All those features make it an excellent candidate for ultraviolet (UV) luminescence devices, light-emitting diodes (LEDs), gas sensors, solar cells, photocatalysis, and biosensors. Especially, doped ZnO with appropriate transition metals (TM) such as Co (Liang et al. 2009), Ni (Hou et al. 2010), Mn (Rekha et al. 2010), Fe (Xu and Li 2010), and Cu (Khan and Ghosh 2011) has attracted much attention because of its potential applications in the areas of photonics, optoelectronics, spintronics, and sensors.

Among TM elements, the Cu dopants have attracted much interest for potential applications in semiconductor devices. Theoretical study indicates that high concentration of Cu can be incorporated into ZnO because of its high ionization energy and the low formation energy of substitutional group-IB elements, and Cu doping can significantly affect the electrical properties, chemical properties, and surface modification of ZnO by creating localized impurity levels and narrowing the band gap of ZnO (Garces et al. 2002; Yan et al. 2006; Xing et al. 2011). Furthermore, Cu behaving as an acceptor in ZnO crystals makes it a good candidate for creating p-type ZnO (Lupan et al. 2011). Expertical results reveal that Cu doping can improve the optical property of ZnO. Shi et al. (2011) have prepared Cu/ZnO catalyst by using sol–gel auto-combustion method, and the catalyst can applicate in low-temperature methanol synthesis. Kanade et al. (2007) reported that self-assembled aligned Cu-doped ZnO nanoparticles showed excellent photocatalytic activity under visible light irradiation. Moreover, Wang and Lin (2011) synthesized Cu-doped ZnO nanoparticle sheets with both violet and yellow emissions, which are promising for white LED applications. Although the optical property of ZnO is significantly improved by Cu doping, the enhancement is still limited for poor photon absorption of ZnO. Thus, it is necessary to further improve the optical properties of Cu-doped ZnO.

With the development in the improvement of the optical property of ZnO, researches on hybrid materials of ZnO and carbon nanotubes (CNTs) have received extensive attention (Baibarac et al. 2008; Liu et al. 2006; Chen et al. 2006), because of the unique internal structure, high surface area, low mass density, remarkable chemical stability, and electronic conductivity of CNTs. As a catalyst carrier, CNTs can not only act as photosensitizers for semiconductor ZnO, but also hinder the recombination of electrons and holes (Eder 2010). Moreover, CNTs have a large surface area, and can act as a dispersing agent that prevents ZnO nanoparticles from agglomerating, resulting in providing a higher active surface area for the resultant catalyst compared with the ZnO nanoparticles. At present, CNTs are widely employed to enhance the photocatalytic activity of ZnO (Zhu et al. 2009) and TiO_2_ (Leary and Westwood 2011; Woan et al. 2009). Hence, it can be foreseen that the optical properties of Cu-doped ZnO is improved in the presence of CNTs, and have potential applications in optoelectronics and photocatalysis.

In our previous study, we successfully coated ZnO nanoparticles on the surface of MWNTs through co-precipitation method after MWNTs were modified with concentrated ammonia and citric acid (Chen et al. 2006). In this article, a simple and efficient approach of treated MWNTs is developed for the synthesis of Cu-doped ZnO nanoparticles on MWNTs. MWNTs are treated by sodium hydroxide and acid, and then Cu-doped ZnO nanoparticles are decorated on the surface of MWNTs. Furthermore, the optical property of MWNTs/Cu-doped ZnO composite powder is studied.

## Experimental

### Preparation and treatment of MWNTs

As-prepared MWNTs (diameters 20–50 nm) were prepared by the chemical catalytic vapor decomposition (CVD) process. The details of the MWNTs preparation have been in the literature (Chen et al. 2002). In a typical treatment, 5 g as-prepared MWNTs were dispersed in 500 mL sodium hydroxide (2 mol/L) and refluxed at boiling for 2 h under stirring. After rinsed with deionized water until the pH value of solution close to neutral, the NaOH-treated MWNTs were dried at 80 °C. In order to remove impurities, these NaOH-treated MWNTs were further oxidized by immersing in a 3:1 mixture of concentrated H_2_SO_4_ and HNO_3_ and refluxing for 2 h at boiling point, subsequently suspending and refluxing in HCl solution for 2 h at the same temperature. Finally, the MWNTs were dried at 80 °C after being filtered and washed with deionized water.

### Preparation of MWNTs/Cu-doped ZnO composite powder

Coating MWNTs with ZnO-based nanoparticles was performed typically as follows: 0.439 g Zn(CH_3_COO)_2_·2H_2_O and Cu(CH_3_COO)_2_·2H_2_O (Cu/Zn = 5 % in molar ratio) were first dissolved in anhydrous ethanol of 100 mL, and then 0.02 g above-treated MWNTs were added into under sonicating for about 15 min. Subsequently, the mixture solution, which composes of oxalic acid and anhydrous ethanol of 100 mL, was slowly dropped into the mixture solution of zinc acetate and copper acetate while stirring at 60 °C, and a sol was produced. Thirdly, the sol was maintained at 80 °C for 48 h to form the precursor. Finally, the above-prepared precursor was annealed at 450, 550, and 650 °C, respectively, for 2 h under the protection of nitrogen. For comparison, the MWNTs/ZnO composite powder was prepared under the same conditions.

### Characterization

Thermogravimetric analysis (TGA) data and differential scanning calorimeter (DSC) data were recorded with a DT-40 Shimadzu thermal analyzer in the range of 25–1,000 °C under nitrogen flow at a rate of 10 °C per minute. X-ray diffraction (XRD) measurements were performed using Philips PW 1710 diffractometer with Cu Kα_1_ radiation. Scanning electron microscopy (SEM) observations and energy dispersive X-ray spectroscopy (EDS) were carried out with a S-4800 field emission scanning electron microscope. Transmission electron microscopy (TEM) analyses were conducted on a H800 transmission electron microscope. UV absorption spectra of the samples in anhydrous ethanol were recorded by TU-2550 spectrophotometer. Fluorescence spectra measurements were characterized for anhydrous ethanol on a Hitachi F4500 fluorescence spectrophotometer at room temperature.

## Results and discussion

### TG and DSC analysis

**Fig. 1:**
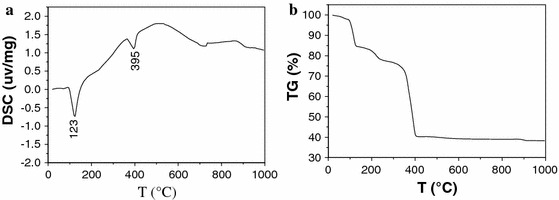
DSC (**a**) and TG (**b**) of the precursor

In order to determine the optimal temperature of heat treatment, the precursor was thermodynamically analyzed by the DSC and TG methods, and the result is presented in Fig. 1. From the DSC curve (Fig. 1a), it can be seen that there are two endothermic peaks at 123 and 395 °C, respectively. The endothermic peak at 123 °C might stem from the loss of crystalloid water and ethyl acetate, and that at 395 °C is attributed to the decomposition of oxalic (copper oxalic and zinc oxalic). The TG curve of the precursor reveals that the weight of the precursor continuously reduced below about 400 °C, while the weight of the precursor remained constant more than 400 °C, as shown in the Fig. 1b. These results indicate that the precursor full converts into the crystallized oxides above 400 °C.

### XRD analysis

**Fig. 2:**
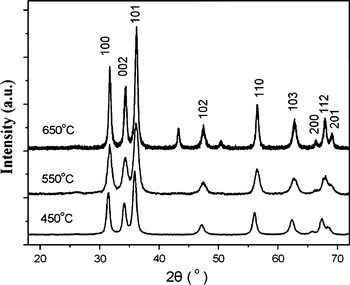
XRD patterns of the MWNT/Cu-doped ZnO composite powder

Figure 2 shows the XRD of MWNTs/Cu-doped ZnO composite powder subjected to heat treatment at different temperatures. It is found that all samples exhibit the diffraction peaks of ZnO corresponding to (100), (002), (101), (102), (110), (103), (200), (112), and (201) planes, respectively. All diffraction peaks of the products are in good agreement with those of the hexagonal wurtzite structure of ZnO (JCPDS card 36-1451). Comparison with three curves, the diffraction peaks become sharper and narrower with increasing temperature of heat treatment, which indicates that the crystallite size increases along with the rise of temperature of heat treatment. Furthermore, no trace of copper metal, oxides, or any binary zinc copper phases is observed in the XRD pattern of samples calcined at 450 and 550 °C. However, the XRD pattern of sample calcined at 650 °C shows that there are two new diffraction peaks at about 2θ = 43.09° and 50.43°, respectively, which are ascribed to (111) and (200) planes of Cu metal. This result suggests that the composite contains Cu element.

### Electron microscopy studies

**Fig. 3:**
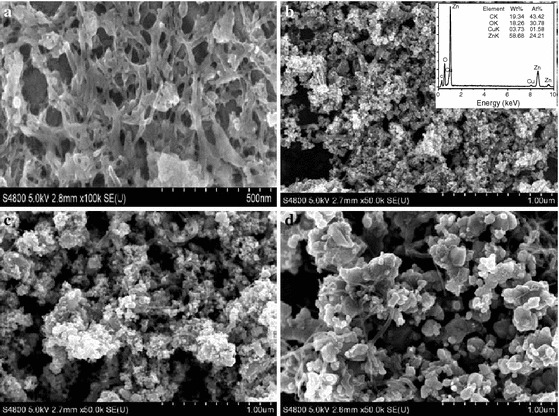
SEM images of the precursor (**a**) and MWNT/Cu-doped ZnO composites powder annealed at different temperature: **b** 450 °C, **c** 550 °C, and **d** 650 °C

SEM images of different samples are shown in Fig. 3. From the SEM image of the precursor, we can see that the surface of the MWNTs is adopted by a layer, as shown in Fig. 3a. After the precursor is annealed at 450 °C, the surface of MWNTs is decorated a layer nanoparticles with the diameter ranged from 10 to 20 nm (shown in Fig. 3b). In order to confirm the element present in sample, EDS is carried out. The results are shown in the inset in Fig. 3b. It reveals the presence of Zn, Cu, O, and C, which indicates that the surfaces of MWNTs are decorated by Cu-doped ZnO nanoparticles, consistent with the results of XRD. Figure 3c reveals the SEM image of MWNTs/Cu-doped ZnO composites after heat treatment of 550 °C. It can be observed that the MWNTs are also continuously coated by nanoparticles. However, the nanoparticle size increases compared with the materials annealed at 450 °C. Figure 3d shows the SEM image of MWNTs/Cu-doped ZnO composite powder annealed at 650 °C. It is evident that the particle size is larger, and many MWNTs protrude from the particles.

**Fig. 4:**
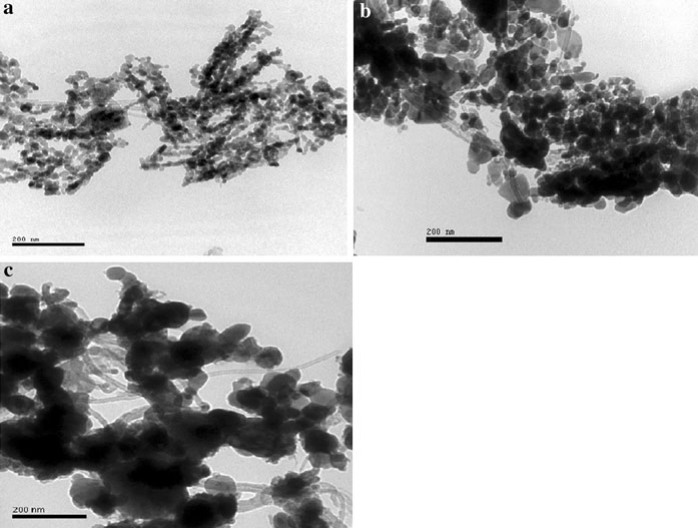
TEM images of MWNT/Cu-doped ZnO composite powder annealed at different temperature: **a** 450 °C, **b** 550 °C, and **c** 650 °C

TEM studies further confirm the success of the attachment of Cu-doped ZnO nanoparticles to the walls of MWNTs, as shown in Fig. 4. Figure 4a shows the TEM morphology of the sample annealed at 450 °C. It is obvious that the MWNTs are uniformly modified by Cu-doped ZnO nanoparticles with sizes of about 10–20 nm. Figure 4b presents the TEM image of the sample annealed at 550 °C. We can see that the MWNTs are also modified by Cu-doped ZnO nanoparticles with sizes of about 50–70 nm. However, the particle size of Cu-doped ZnO particles increases to about 100 nm at 650 °C (as shown in Fig. 4c), which further confirms the results of the XRD and SEM.

### Studies of optical properties

**Fig. 5:**
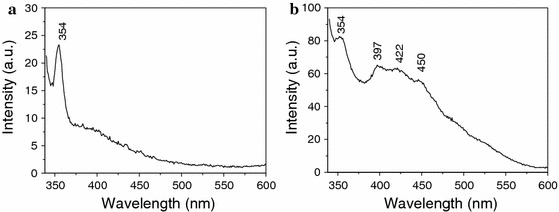
Fluorescence spectrum of MWNT (**a**) and MWNT/ZnO composite annealed at 450 °C (**b**)

Figure 5 shows the room-temperature fluorescence spectrum of MWNTs and MWNTs/ZnO composite powder in which the wavelength of excitation is 320 nm. It is clear that there is a UV peak at about 354 nm on the spectrum of MWNTs (shown in Fig. 5a), while the fluorescence spectrum of MWNTs/ZnO composite powder (Fig. 5b) shows that there are four fluorescence peaks at 354, 397, 422, and 450 nm, respectively. The peak at about 397 nm is assigned to UV emission originating from free excitation emission at about 3.37 eV from the wide band gap of ZnO. The two blue emission peaks at 422 and 450 nm are ascribed to the Zn interstitial (Zni) and Zn vacancy (V_Zn_) level transition. The UV emission peak at about 354 nm is attributed to MWNTs, which may come from the trapping of excitation energy at defect sites of MWNTs and containing extended *ð*-electronic structures in the surface of modified MWNTs (Liang et al. 2000).

**Fig. 6:**
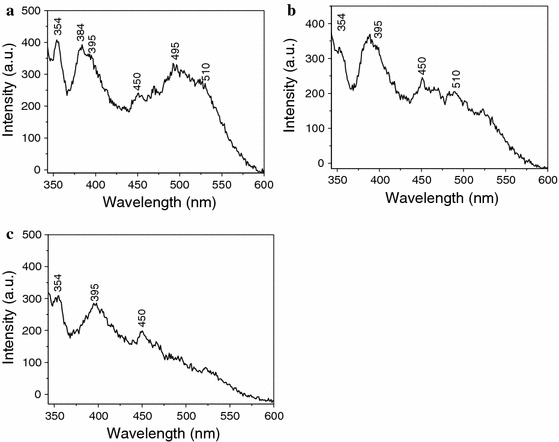
Fluorescence spectrum of MWNT/Cu-doped ZnO composite powder annealed at different temperature: **a** 450 °C, **b** 550 °C, and **c** 650 °C

The room-temperature fluorescence spectrum of MWNTs/Cu-doped ZnO composite powder annealed at different temperature is shown in Fig. 6. As we can see, all the spectra have several peaks locating at the wavelength of ~354, ~380–395 and 450 nm, respectively, and a green emission band centered at ~495–510 nm is observed in the spectrum of MWNTs/Cu-doped ZnO composite powder annealed at 450 and 550 °C. Moreover, it is very obvious that the intensity of green emission peak decreases with increasing the temperatures of heat treatment.

It is generally accepted that the green emission derives from the single ionized oxygen vacancy in the ZnO, and the emission peak results from the radiative recombination of electrons in singly occupied oxygen vacancies with photoexcited holes in the valence band (Yan et al. 2007). Cu mixes into the sublattice of ZnO, resulting in producing more defects in ZnO (Persson et al. 2006). Two mechanisms about Cu-doped ZnO have been proposed. One is that copper replaces the zinc site of ZnO lattice. When the Zn^2+^ site is substituted by Cu^2+^ (Herng et al. 2007; Lupan et al. 2010), there will form a defect bond in solid state lattice of ZnO between 3d copper and 2p oxygen orbitals, and create a single-acceptor state above valence band E*v* of ZnO, resulting in narrowing the band gap of ZnO (Yan et al. 2006). Another is that Cu inserts into the Zni. The lattice of ZnO is distorted after the Cu inserts into the Zni, leading to forming oxygen vacancies and zinc vacancies. Hence, the intensity of green emission peak is significantly improved by Cu doping. However, this configuration of Cu has low thermal stability, and increased annealing temperature leads to partial Cu outdiffusion. According to literature (Wahl et al. 2004.), when annealing temperature is lower than 600 °C, a large fraction of Cu atoms (60–70 %) occupy almost ideal substitutional Zn sites, while annealing above 600 °C causes partial Cu outdiffusion. Furthermore, the ZnO crystallite becomes better as the temperatures of heat treatment increase. Cu outdiffusion and better crystallite will restore the defects and decrease the oxygen vacancy concentration in the ZnO crystal, therefore the intensity of green emission peak decreases with increasing the temperatures of heat treatment.

**Fig. 7:**
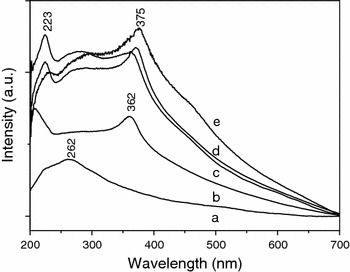
UV absorption spectrum of different samples: *a* pure MWNT, *b* MWNTs/ZnO composite annealed at 450 °C, *c* MWNTs/Cu-doped ZnO composite annealed at 450 °C, *d* MWNTs/Cu-dopedZnO composite annealed at 550 °C, and *e* MWNTs/Cu-doped ZnO composite annealed at 650 °C

Figure 7 displays the UV–Vis absorption spectrum of MWNTs and composite powder in anhydrous ethanol. Curve a in Fig. 7 is the UV–Vis absorption spectrum of pure MWNTs. A very broad absorption peak appears at about 262 nm, which originates from the C=C structure of MWNTs. From the UV spectrum of MWNTs/ZnO composite powder, we can see that there are two UV absorption peaks at about 210 and 362 nm, respectively, as shown in the curve b of Fig. 7. The two absorption peaks are attributed to MWNTs and the characteristic peaks of ZnO, respectively. The spectra of all MWNT/Cu-doped ZnO composite powder show two sharp absorption peaks at about 223 and 375 nm, which have a slight red shift with respect to the MWNTs/ZnO composite powder. Similar red shift in Cu-doped ZnO was also reported (Ferhat et al. 2009; Bylsma et al. 1986; Reddy et al. 2011), which is attributed to the strong p-d mixing of O and Cu. Furthermore, it is a very interesting phenomenon that the samples of MWNT/Cu-doped ZnO composite powder exhibit a broad absorption bands from 200 to 400 nm, and its absorption intensity is stronger than that of MWNT/ZnO composite powder, indicating the effective photoabsorption property for this doped composite powder.

**Fig. 8:**
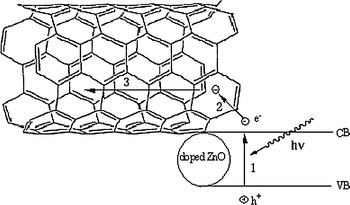
The complete scheme of transfer electron

From the above results, it is concluded that the UV absorption ability of ZnO is significantly improved through adding the MWNTs and doping Cu in ZnO, which comes from the outstanding electronic property of MWNTs. MWNTs have a large electron-storage capacity (one electron for every 32 carbon atoms), and may accept photon-excited electrons in mixtures or nanocomposites (Kongkanand and Kamat 2007). Furthermore, MWNTs may play a crucial role in the charge transport (Eder 2010). There are three processes about the electron transport in the MWNTs/Cu-doped ZnO composite, as shown in Fig. 8. Firstly, when a high-energy photon excites an electron from the valence band of the ZnO, the photogenerated electron formed in the space-charge regions is transferred into the conduction band of the ZnO. Secondly, MWNTs capture the photon-excited electrons from the conduction band of the ZnO. Thirdly, the captured electron can fast be conducted by MWNTs, leaving a hole in the valence band of the ZnO.

## Conclusion

Cu-doped ZnO nanoparticles were successfully decorated onto the surface of MWNTs through the co-precipitation method. Because of adding MWNTs and Cu, a new UV emission peak at about 354 nm and a green emission band at about 510 nm are observed in the spectrum of ZnO, and the UV absorption ability of ZnO is significantly improved, which may improve the utilization rate of the visible light. Moreover, MWNTs can act as photosensitizers for n-type semiconductors and hinder the recombination of electron–hole pairs, resulting in improving ZnO photocatalytic activity. Accordingly, the synthetic products have a potential application in fields of photocatalysis and optoelectronics.
